# Fermentation of *Propionibacterium acnes,* a Commensal Bacterium in the Human Skin Microbiome, as Skin Probiotics against Methicillin-Resistant *Staphylococcus aureus*

**DOI:** 10.1371/journal.pone.0055380

**Published:** 2013-02-06

**Authors:** Muya Shu, Yanhan Wang, Jinghua Yu, Sherwin Kuo, Alvin Coda, Yong Jiang, Richard L. Gallo, Chun-Ming Huang

**Affiliations:** 1 Division of Dermatology, Department of Medicine, University of California San Diego, La Jolla, California, United States of America; 2 Sanford-Burnham Institute for Medical Research, La Jolla, California, United States of America; 3 Surface Bioadvances Inc., San Diego, California, United States of America; 4 Moores Cancer Center, University of California San Diego, La Jolla, California, United States of America; University of Padova, Medical School, Italy

## Abstract

Bacterial interference creates an ecological competition between commensal and pathogenic bacteria. Through fermentation of milk with gut-friendly bacteria, yogurt is an excellent aid to balance the bacteriological ecosystem in the human intestine. Here, we demonstrate that fermentation of glycerol with Propionibacterium acnes (P. acnes), a skin commensal bacterium, can function as a skin probiotic for in vitro and in vivo growth suppression of USA300, the most prevalent community-acquired methicillin-resistant Staphylococcus aureus (CA-MRSA). We also promote the notion that inappropriate use of antibiotics may eliminate the skin commensals, making it more difficult to fight pathogen infection. This study warrants further investigation to better understand the role of fermentation of skin commensals in infectious disease and the importance of the human skin microbiome in skin health.

## Introduction

Bacterial interference, or bacteriotherapy, in which commensal bacteria are used to prevent colonization of the host by pathogens, has been shown to be a promising modality for prevention and treatment of infections [1], [2]. Yogurts containing live probiotic strains, the best examples of bacterial interference, have been used for centuries to maintain the digestive microbial ecosystem. Bacterial interference via fermentation commonly takes place in natural ecosystems as well. For instance, microorganisms both on and inside fruits metabolize sugars to produce fermentation products including short-chain fatty acids (SCFAs) during ripening. These fermentation products have been found to inhibit activity of bacterial competitors within ripe fruit [3]. Although not identical microenvironments, reports show that SCFAs produced by fermentation of microorganisms have been detected in pus from deep-seated abscesses, anaerobic microenvironments in the context of human bacterial infection [4].

Each individual carries approximately ten times more bacterial cells than human cells [5]. The skin is the human body’s largest organ, colonized by a diverse milieu of microorganisms (skin microbiome), most of which are commensals as they are harmless and even beneficial to their host. Propionibacterium acnes (*P. acnes*) is a predominant bacterium in skin microbiome [5], [6]. Everyone hosts *P. acnes* [5], [6] which accounts for approximately half of the total skin microbiome, with an estimated density of 10^2^ to 10^5–6^ cm^2^ [7]–[9]. *Staphylococcus aureus* (S. aureus) is a Gram-positive bacterium and one of the major causes of both hospital- and community-acquired infections worldwide. This bacterium is responsible for a broad range of infections such as skin wound and disseminated systemic infections leading to organ failure and death [10]. It is estimated that *S. aureus* accounts for 12 million outpatient visits and 292,000 hospitalizations of which 126,000 are due to methicillin-resistant *S. aureus* (MRSA) annually in the United States (US) alone [11]. The Center for Disease Control Prevention (CDC) estimates that more than 90,000 people die from hospital-acquired bacterial infections in US each year [12]. Community-acquired MRSA (CA-MRSA) is reported as the most common cause of purulent skin and soft tissue infections in the US [13]. These infections are particularly difficult to treat because the bacterium has become resistant to many commonly used antibiotics.

Previous studies indicated that *P. acnes* was found in the human skin wounds [14]. *P. acnes* and *S. aureus* were co-isolated from both shoulder sepsis [15] and prosthetic hip infections [15], [16] in adult patients. Although *P. acnes* predominates on the skin surface and is exposed to atmospheric oxygen, we hypothesize that *P. acnes* enters the dermis when a deep wound is created by pathogen infection. The anaerobic microenvironment in deep-seated abscesses may trigger P. acnes to undergo fermentation using carbon sources (such as glycerol and glucose) naturally produced in skin. Human hosts exploit P. acnes fermentation in deep-seated abscesses to prevent the entry of S. aureus into the bloodstream and reduce the risk of systemic S. aureus infections.

Propionibacterium acnes was so-named for its ability to ferment carbohydrates to propionic acid, a SCFA known to have antimicrobial activity [17], [18]. Our results demonstrated that P. acnes can fermentatively metabolize glycerol, an endogenous skin metabolite, in mouse skin. The fermentation products of *P. acnes* significantly suppress the *in vitro* and *in vivo* growth of USA300, a CA-MRSA. These results show that fermentation of *P. acnes* in the human skin microbiome may play a role in human innate immunity against *S. aureus*. The use of *P. acnes* fermentation for bacterial interference therapy leverages evolutionary medicine by lowering selective pressure for antibiotic resistance and developing therapeutics with negligible side effect profiles.

## Results

### *P. acnes* Fermentation Counteracts USA300

**Figure 1:**
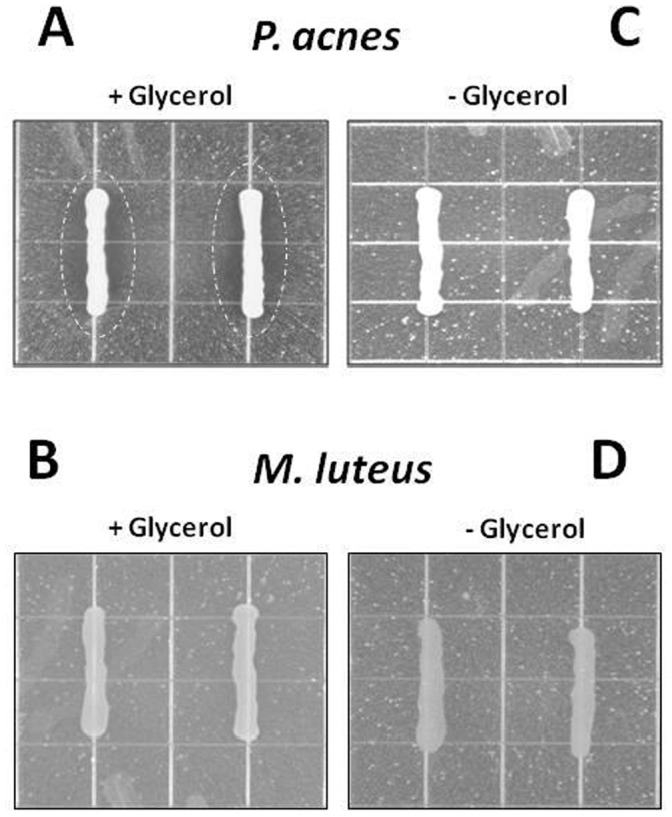
*P. acnes* interferes with the growth of USA300 in the presence of glycerol. An overlay assay reveals zones (circles) of inhibition of USA300 growth when *P. acnes* (ATCC6919, 10^5^ CFU) (A), not *M. luteus* (B), was grown with USA300 in the presence of glycerol in agar plates under anaerobic conditions at 30°C. No inhibition zones were developed when *P. acnes* (C) or *M. luteus* (D) was grown with USA300 in the absence of glycerol.

To examine if *P. acnes* fermentation affects the growth of USA300, *P. acnes* or Micrococcus luteus (M. luteus), a Gram-positive, non-fermenting skin commensal bacterium, was grown on agar plates in the presence or absence of glycerol, a naturally occurring metabolite found in human skin [19], for three days before growing USA300 in the overlaid agar. As shown in Figure 1A, only *P. acnes* grown with glycerol showed visible inhibitory effects against USA300. No inhibitory effect was observed when *P. acnes* was grown in the absence of glycerol (Figure 1C). M. luteus grown with or without glycerol did not display inhibitory effects against the growth of USA300 (Figure 1B and D). These findings suggest that glycerol fermentation is required for inhibitory effect of *P. acnes* against USA300.

**Figure 2:**
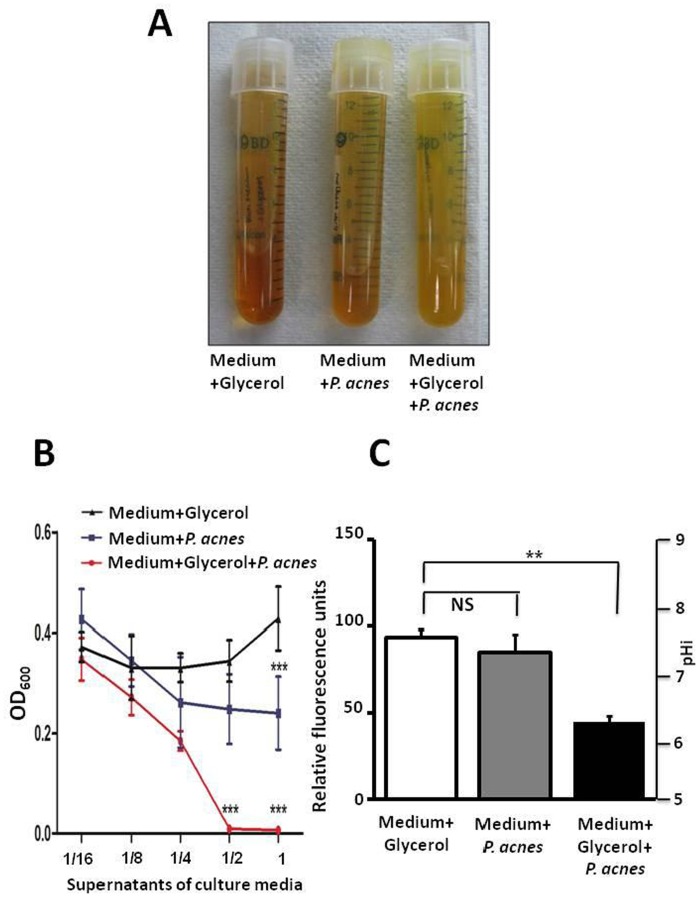
Probiotic effects of *P. acnes* fermentation against USA300 accompanied by a decrease in intracellular pH. (A) P. acnes (10^5^ CFU/ml), was incubated in rich medium in the absence (▪) and presence (•) of glycerol under anaerobic conditions for ten days. Rich medium plus glycerol without P. acnes (▴) was included as a control. (B) After a 17-day incubation, fermented or control media were then collected, diluted (1/2 to 1/16) and added to cultures of USA300 (10^5^ CFU/ml) overnight. The inhibitory growth of USA300 was defined as a decline in OD_600_. (C) The cFSE-loaded USA300 (3×10^4^ CFU) was treated with 100 µl fermented (Medium+glycerol+P. acnes; solid bar) or control [(Medium+glycerol (open bar) and Medium+P. acnes (grey bar)] media. The change in the relative fluorescence units corresponding to intracellular pH of USA300 was measured 5 min after treatment. **P<0.01; ***P<0.001 (two-tailed t-tests). Data are the mean ± standard deviation (SD) of three individual experiments. NS: Non-significant.

To test the probiotic effect of *P. acnes* fermentation products, *P. acnes* was incubated in rich medium under anaerobic conditions in the presence of glycerol as the carbon source. Rich medium plus glycerol and rich medium plus *P. acnes* were used as controls. To monitor the fermentation process, cultures were tested with phenol red, a fermentation indicator, to assess SCFA production as a result of glycerol fermentation. Only media in the culture of *P. acnes* with glycerol turned yellow (more acidic) ten days following incubation (Figure 2A), demonstrating *P. acnes* fermentation. This was further validated quantitatively by pH values in rich medium containing glycerol, *P. acnes* and glycerol plus *P. acnes* of 6.6, 6.5, and 5.6 respectively, following 17 days of incubation. To assess the anti-CA-MRSA activity of *P. acnes* fermentation products, USA300 [1×10^5^ colony-forming unit (CFU)/ml] was then incubated with each group and their respective serial two-fold dilutions (1/2 to 1/16) overnight. Initial fermented media and its ½ dilution markedly suppressed the growth of USA300 (Figure 2B).

### Ferments of *P. acnes* Reduce the Intracellular pH of USA300

SCFAs are the principal end products of bacterial fermentation [20]. The antimicrobial effects of SCFAs result mainly from the un-dissociated form of SCFAs [21], [22]. Non-dissociated SCFAs passively diffuse through the cell wall of bacteria. Once internalized into the neutral pH of the cell cytoplasm, they can dissociate into anions and protons. Elevation of both anions and protons is a potentially lethal factor to bacteria that must maintain a near neutral pH cytoplasm to sustain functional macromolecules. Export of excess protons requires consumption of cellular adenosine triphosphate (ATP) and may result in depletion of cellular energy [21]. To determine the action mechanism of fermentation products of *P. acnes* against CA-MRSA, we loaded USA300 with an internally conjugated fluorescent pH probe, carboxyfluorescein succinimidyl ester (cFSE). Compared to control media, fermented media of *P. acnes* significantly lowered the intracellular pH of USA300 (Figure 2C), consistent with previous findings that a lowered intracellular pH of bacteria is a lethal mechanism of SCFA [22].

### Identification of SCFAs in Fermentation Products of *P. acnes* by Nuclear Magnetic Resonance (NMR) Analysis

To identify the SCFAs in bacterial fermentation, *P. acnes* (ATCC6919) was incubated in rich media under anaerobic conditions in the presence of ^13^C_3_-glycerol (20 g/l) for 17 days. The ^13^C-labeled metabolites in the fermentation products of *P. acnes* were identified by NMR analysis. In addition to un-metabolized ^13^C-glycerol (62.3 and 72.5 ppm), three SCFAs (acetic acid, lactic acid, and propionic acid) were detected in the fermentation productions of *P. acnes*. In a one-dimensional (1-D) ^13^C NMR spectrum using an NMR spectrometer (400 MHz JEOL JNM-ECS) (Figure S1A), two signals at 21.2 and 69.5 ppm corresponded to ^13^C-labeled lactic acid. Two ^13^C-labeled propionic acids appeared at 8.9 and 31.0 ppm. Acetic acid was detected at 24.3 ppm. The signals of acetic acid (1.90 ppm), lactic acid (1.33 and 4.09 ppm), and propionic acid (1.15 and 2.38) in a 1-D ^1^H NMR spectrum were displayed in Figure S1B. Three major SCFAs including propionic acid in the fermentation products of *P. acnes* were shown in a two-dimensional (2-D) ^1^H-^13^C HSQC NMR spectrum (Figure S1C). Propionibacterium including *P. acnes* produces propionic acid as the end product of its anaerobic fermentation [23]. Thus, the results above demonstrate that *P. acnes* fermentatively metabolizes ^13^C_3_-glycerol into SCFAs.

### Fermentation Products of *P. acnes* Suppress USA300-infected Lesions and Bacterial Colonization

**Figure 3:**
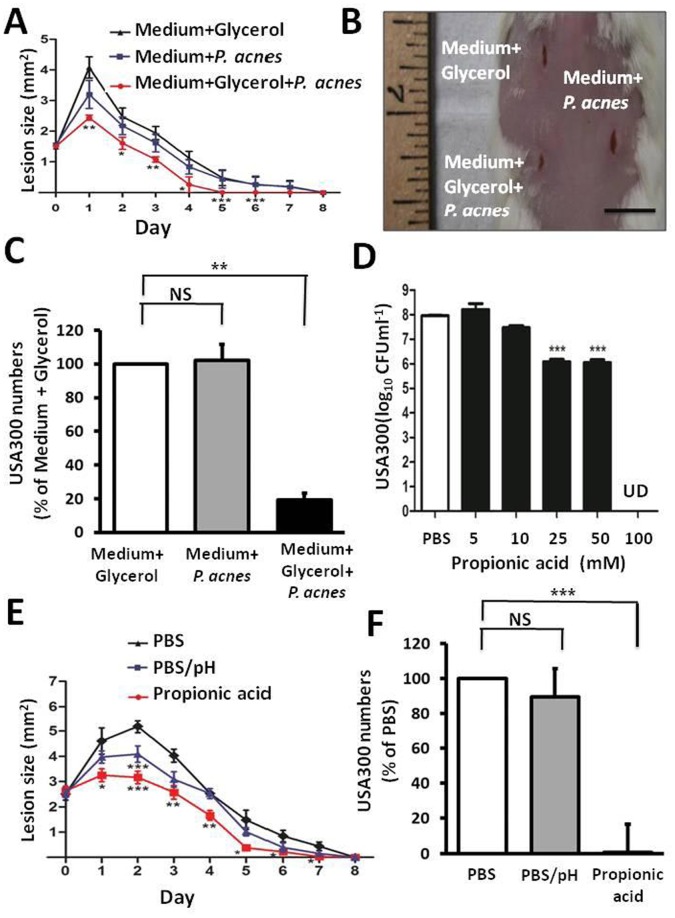
Fermented media of *P. acnes* and propionic acid suppress the infection of USA300 in mouse skin. (A) USA300 bacteria (2×10^6^ CFU) were applied onto the wounded areas 10 min after application of culture supernatants of *P. acnes* in the absence and presence of glycerol or a control (medium plus glycerol). The lesion size was recorded daily for 8 days. (B) Skin lesions pictured on day 1 after bacterial application were illustrated. Bar = 1 cm. (C) Three days after bacterial application, the USA300 numbers in the skin were enumerated and presented as % of control. (D) For MBC assays, USA300 (10^6^ CFU/ml) was incubated with propionic acid (5–100 mM in PBS) in media on a 96-well microplate overnight. Bacteria incubated with PBS alone served as a control. After incubation, USA300 was diluted 1∶10–1∶10^6^ with PBS, and 5 µl of the dilutions were spotted on an agar plate for CFU counts. **P<0.01; ***P<0.001 (two-tailed t-tests). Data are the mean ± SD of three individual experiments. UD, undetectable. (E) USA300 bacteria (2×10^6^ CFU) were applied onto the wounded areas 10 min after application of propionic acid (5 µl; 100 mM) or PBS (5 µl). The lesion size was measured daily for 8 days. *P<0.05; **P<0.01; ***P<0.001 (A, E). (F) Three days after bacterial application, the USA300 numbers in the skin were counted and presented as % of PBS control. **P<0.01; ***P<0.001 (C, F). *P*-values were evaluated using two-tailed *t*-tests. Data are the mean ± SD of lesions from three separate experiments performed with five mice per group. NS: Non-significant.

Although *S. aureus* can cause a life-threatening and systemic infection (bacteremia), skin and soft tissue are the most common sites of *S. aureus* infection and comprise more than 75% of MRSA disease [24]. Eradication of infected *S. aureus* in skin will prevent the bacteria entering the bloodstream. To mimic the natural route of CA-MRSA infection, a 5 mm wound was made on the dorsal skin of Institute of Cancer Research (ICR) mice and USA300 bacteria were topically applied onto the wound. To test the potency of *P. acnes* fermentation products against CA-MRSA *in vivo*, we inoculated USA300 (2×10^6^ CFU) to skin wounds 10 min after topical application of *P. acnes* ferments or controls. Application of fermented media, but not controls, considerably decreased the sizes of USA300-infected skin lesions (Figure 3A and B). To determine the intensity of bacterial colonization, wounds were homogenized to estimate the CFU. The USA300 counts in wounds applied with culture supernatants of medium plus glycerol, medium plus *P. acnes* or medium plus glycerol and *P. acnes* were 4.8±0.9×10^6^, 4.9±2.6×10^6^ and 9.4±4.5×10^5^ CFU/g, respectively. These result show that application of fermented media of *P. acnes* decreased 80% of USA300 colonization in the lesions as compared to application of control media (Figure 3C).

To examine if propionic acid exerts antimicrobial activity against CA-MRSA, we determined the minimal bactericidal concentration (MBC) value of propionic acid for USA300. Bacteria were incubated with propionic acid at various concentrations in their media overnight at 37°C. After incubation, the bacteria were diluted with PBS and spotted on an agar plate to count CFU. We found that propionic acid effectively suppressed the growth of USA300 (Figure 3D) at a concentration greater than 25 mM and completely killed all of bacteria at a concentration greater than 100 mM. To assess the antimicrobial activity of propionic acid *in vivo*, USA300 (2×10^6^ CFU) was inoculated to the wounded areas of ICR mice 10 min following topical application of either propionic acid (100 mM), PBS (pH 7.1) or acidic PBS (pH 3.5, corresponding to pH for 100 mM propionic acid). Application of propionic acid significantly reduced the size of USA300-infected skin lesions as compared to application of both PBS solutions (Figure 3E). On Day 1, the sizes of USA300-infected skin lesions in PBS (pH 7.1)-, acidic PBS (pH 3.5)-, or propionic acid-treated wounds were 4.6±0.6, 4.0±0.8, and 3.2±0.8 mm^2^, respectively. A significant decrease in the size of USA300-infected skin lesion was detected two days following application of PBS (pH 3.5). Histological observation [hematoxylin and eosin (H&E) staining] revealed that the propionic acid-treated wounds had an attenuated inflammatory response and improved integrity of epidermis and hair follicle compared to PBS-treated wounds (Figure S2). This suggests that propionic acid reduced USA300-induced damage of the epidermal layers and inflammation. To determine the bacterial colonization, USA300-infected skin lesions treated with propionic acid and controls were homogenized to estimate CFU. The bacterial numbers in lesions were: 1.9±1.0×10^9^ (PBS, pH 7.1), 1.7±1.5×10^9^ (PBS, pH 3.5) and 1.4±1.4×10^7^ (propionic acid) CFU/g (Figure 3F), suggesting that propionic acid significantly decreased the growth of USA300 in the skin lesions.

### Fermentation of *P. acnes* Occurs in Mouse Skin

**Figure 4:**
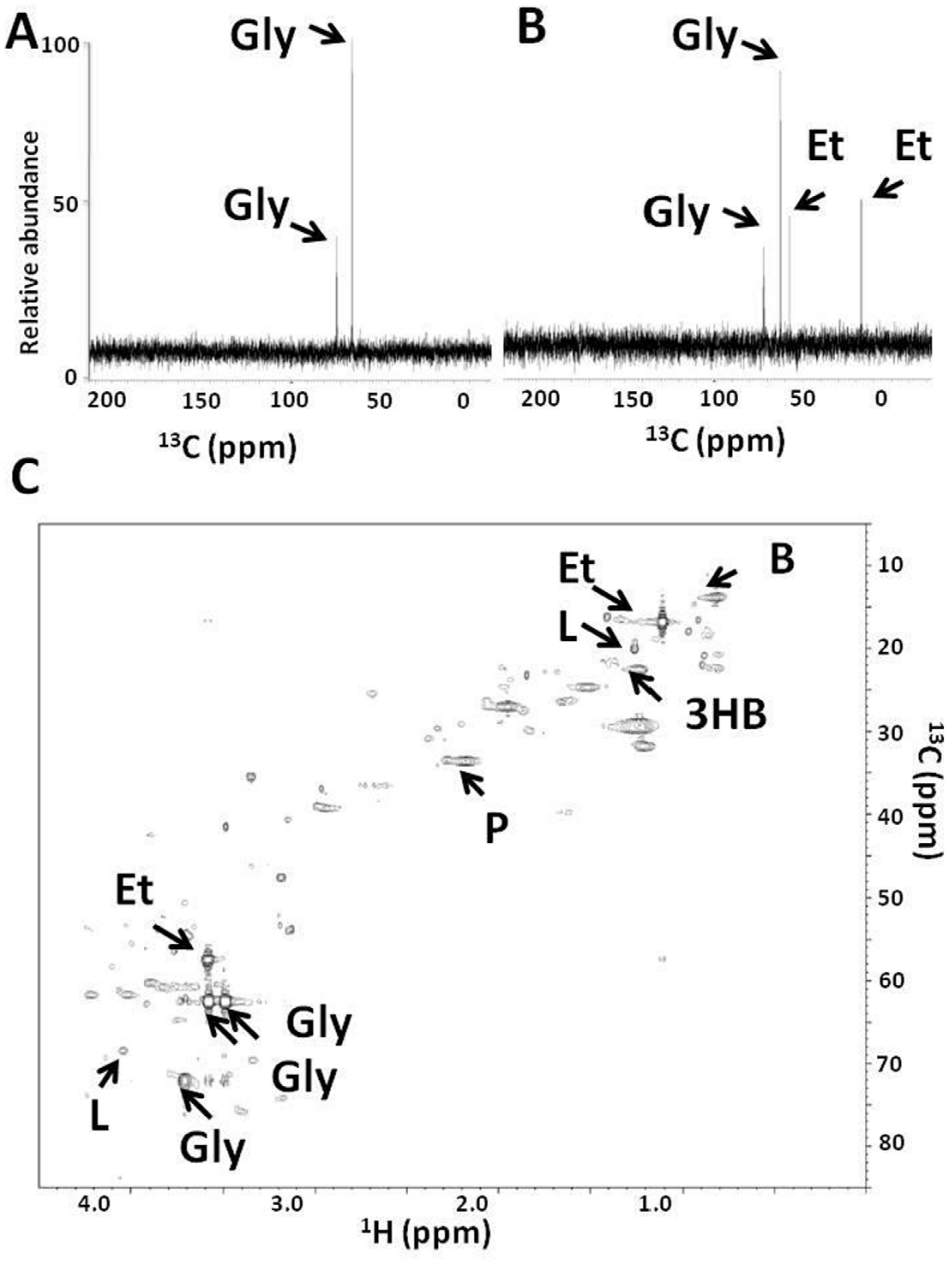
NMR validation of fermentation of *P. acnes* in mouse skin. (B) The ear of ICR mice was intradermally injected with ^13^C_3_-glycerol (0.2 mg) and P. acnes (ATCC6919; 10^7^ CFU in 10 µl PBS) for 3 days. (A) The other ear of the same mouse received ^13^C_3_-glycerol (0.2 mg) and PBS (10 µl) as a control. Supernatants of ear homogenates were mixed with 10% D_2_O and analyzed by a NMR (400 MHz JEOL JNM-ECS) spectrometer. Data from 1,024 scans were accumulated. The NMR signals (17.1 and 58.4 ppm) of ^13^C-ethanol (Et) metabolized from ^13^C_3_-glycerol (Gly) were detected exclusively in the mice injected with ^13^C_3_-glycerol and *P. acnes*. The un-metabolized ^13^C_3_-glycerol appears between 60 and 80 ppm in the ^13^C-NMR spectrum. (C) A 2-D ^1^H-^13^C HSQC NMR spectrum (600 MHz) was displayed. In addition to glycerol (Gly), ethanol (Et), four SCFAs [butyric acid (B), 3-hydroxy-butyric acid (3HB), lactic acid (L), and propionic acid (P)] were detected in the ear injected with glycerol and *P. acnes*.

To date, no evidence demonstrates that bacteria can employ the fermentative process to produce SCFAs *in vivo*. To determine if *P. acnes* is able to ferment carbon sources *in vivo*, ear of ICR mice was intradermally injected with ^13^C_3_-glycerol (0.2 mg) immediately before injection of P. acnes (ATCC6919; 10^7^ CFU in 10 µl PBS). As a control, the same amount of ^13^C_3_-glycerol and PBS was injected to the other ear. One, two and three days after injection, ears were excised, homogenized, and then centrifuged. Supernatants of ear homogenates in 10% deuterium oxide (D_2_O) were subjected to 1-D ^13^C NMR analysis. In addition to ^13^C_3_-glycerol (62.3 and 72.5 ppm), two strong signals of ^13^C-labeled metabolites were detected at 17.1 and 58.4 ppm (Figure 4B) in the mice injected with ^13^C_3_-glycerol plus *P. acnes* for three days. These two signals were undetectable in the mice injected with ^13^C_3_-glycerol plus *P. acnes* for one or two day(s) (data not shown). No ^13^C-labeled metabolites, except ^13^C_3_-glycerol, were detected in the mice injected with ^13^C_3_-glycerol plus PBS (Figure 4A). These two signals at 17.1 and 58.4 ppm correspond to the chemical groups (-CH_3_ and -CH_2_OH) of ethanol. In 1-D ^1^H NMR analysis, two detected signals at 1.15 and 3.64 ppm validate the production of ethanol (data not shown). To identify the SCFAs in the fermentation products, supernatants of ear homogenates in D_2_O were subjected to 2-D ^13^C and ^1^H NMR analysis. In addition to ethanol, four SCFAs (butyric acid, 3-hydroxy-butyric acid, lactic acid, and propionic acid) were detected in the ear injected with ^13^C_3_-glycerol plus *P. acnes* (Figure 4C). The results demonstrate that *P. acnes* is able to undergo fermentation *in vivo*.

### *P. acnes* Fermentation in Skin Wounds Diminishes the Colonization of USA300

**Figure 5:**
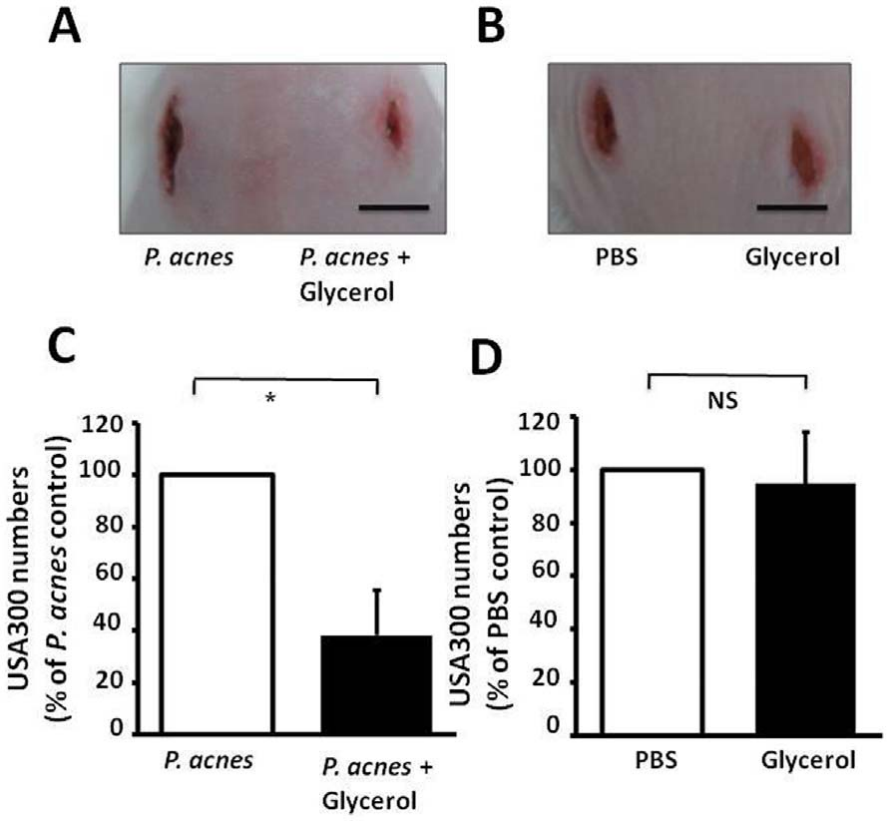
Glycerol fermentation of *P. acnes* in skin wounds diminishes the colonization of USA300. *P. acnes* (10^7^ CFU in 5 µl PBS), P. acnes and glycerol (0.2 mg), PBS (5 µl) alone or glycerol alone were applied onto the skin wounds for 3 days before administration of USA300 (10^7^ CFU in 5 µl PBS) onto the wounded areas. (A, B) Skin lesions pictured 3 days after USA300 application were illustrated. Bar = 0.5 cm. (C) The USA300 numbers in the skin wounds were enumerated 3 days after USA300 application and presented as % of those in skin applied with *P. acnes* (C) or PBS (D). *P<0.05. *P*-values were evaluated using two-tailed *t*-tests. Data are the mean ± SD of lesions from five mice per group. NS: Non-significant.

Although we demonstrated that *P. acnes* grown with glycerol developed inhibition zones against USA300 in an overlay assay *in vitro* (Figure 1), it is unclear if *P. acnes* fermentation in skin wounds can prevent or mitigate a subsequent infection of CA-MRSA. A wound made on the dorsal skin of ICR mice was topically applied with *P. acnes* (10^7^ CFU in 5 µl PBS) or P. acnes and glycerol (0.2 mg) for three days. USA300 (10^7^ CFU in 5 µl PBS) was subsequently administered onto the same wounds for additional three days. Compared to application of *P. acnes* alone, application of P. acnes and glycerol showed improved healing of the USA300-infected skin lesions (Figure 5A). In addition, application of P. acnes and glycerol onto the wounds prior to administration of USA300 reduced more than 50% of USA300 colonization in comparison with application of P. acnes alone (Figure 5C). To investigate the effects of glycerol on the USA300 colonization, PBS (5 µl) or glycerol (0.2 mg) was topically applied to skin wounds for three days followed by a 3-day USA300 infection. Compared to PBS, glycerol did not significantly alter the size of USA300-infected lesions (Figure 5B) and USA300 colonization (Figure 5D). These findings indicate that the suppression of USA300 may be due to glycerol fermentation of *P. acnes* creating an unfavorable environment for USA300, rather than glycerol weakening the defense system of USA300.

## Discussion

Mounting evidence demonstrates that human commensal bacteria may be part of human immunity by exhibiting bacterial interference against pathogens [25]. For example, Staphylococcus epidermidis (*S. epidermidis*), a human commensal bacterium commonly found in the skin, can activate Toll-like receptor 2 (TLR2) signaling and induce antimicrobial peptide expression, thus enabling the skin to mount an enhanced response to pathogens including *S. aureus* [26]. *S. epidermidis* in the nasal cavity can secrete serine protease Esp to inhibit biofilm formation and nasal colonization by *S. aureus* [27]. Here we introduce a novel mechanism by which the skin commensal *P. acnes* makes use of fermentative processes to hinder the colonization of CA-MRSA in the skin wounds. In mouse skin, *P. acnes* can ferment glycerol to various SCFAs including propionic acid (Figure 4). Previous studies indicated that SCFAs in skin play a crucial role in influencing the predominant residence of bacteria on normal human skin [18]. It is known that *P. acnes* and *S. epidermidis* have higher tolerances to propionic acid than other pathogens [28]. In fact, as shown in Figure 3D, propionic acid (25 mM) exerted excellent antimicrobial activity against USA300, but did not affect the growth of *P. acnes* (data not shown). Thus, *P. acnes* fermentation may have a low risk of disrupting the balance of skin microbiome. It has been documented that propionic acid inhibited the various strains (e.g. ATCC29247 [29] and CMCC(B)26003 [30]) of *S. aureus* besides USA300, suggesting the broad spectrum anti-*S. aureus* activity of propionic acid.

The identified ^13^C-labeled SCFAs in Figure 4 were not directly derived from ^13^C_3_-glycerol metabolism by mouse skin cells since no ^13^C-labeled SCFAs were detected in the mice injected with ^13^C_3_-glycerol plus PBS. Furthermore, the presence of ^13^C-labeled propionic acid in the mice injected with ^13^C_3_-glycerol plus *P. acnes* validated that the identified ^13^C-labeled SCFAs resulted from the *P. acnes* fermentation. However, the SCFA profile of ^13^C_3_-glycerol fermentation by *P. acnes in vivo* (Figure 4) was not exactly the same as that *in vitro* (Figure S1). Although propionic acid was measureable in the products of both *in vivo* and *in vitro* glycerol fermentation, ethanol, butyric acid and 3-hydroxy-butyric acid were only detected when ^13^C_3_-glycerol plus *P. acnes* were injected into mice. *P. acnes* HL027PA2 (NCBI Taxon ID 765112) expressed butyryl-CoA dehydrogenase, an enzyme involved in the production of butyric acid during a fermentation process of anaerobic bacteria. A *P. acnes* strain from health cow rumen fluid had the capability to ferment carbohydrates to butyric acid [31]. Some *P. acnes* strains were able to produce butyric acid when they were incubated in culture media containing glucose and tributyrin, a triglyceride which can be converted to butyric acid by *P. acnes* lipase [32]. *P. acnes* (KPA171202) expressed alcohol dehydrogenase [33], a key enzyme for production of ethanol. Despite the presence of ethanol and butyric acid in mouse skin injected with ^13^C_3_-glycerol plus *P. acnes* in this study, the metabolites of glycerol fermentation of *P. acnes in vivo* were not well characterized in the literature. We cannot rule out the possibility that host (e.g. pH in skin) affects the *in vivo* fermentation properties of *P. acnes* [34].

SCFAs can regulate several leukocyte functions including production of cytokines [(tumor necrosis factor (TNF)-α, interleukins (IL)-2, IL-6 and IL-10]. The ability of leukocytes to migrate to the foci of inflammation and destroy microbial pathogens can be affected by SCFAs [28]. In addition, SCFA and butyric acid, in particular, significantly reduce expression of *S. aureus*-induced IL-2 and interferon (IFN)-γ [35]. SCFAs have been demonstrated as ligands for free fatty acid receptor 1 (Ffar1) [also called G-protein-coupled receptor 40 (GPR40)] [36]. Previous studies indicated that GW9508 (GlaxoSmithKline), an arylalkyl derivative of the propionic acid, activated the Ffar1 receptor, suppressed chemokine induction in keratinocytes and attenuated cutaneous immune inflammation [37]. Thus, the use of Ffar1 knockout [Ffar1(−/−) mice may be able to determine if Ffar1 is essential for anti-inflammatory action of SCFAs. As shown in Figures 1 and 5, the inhibitory effect of *P. acnes* on the growth of USA300 was detectable exclusively through glycerol fermentation. Glycerol itself did not influence the growth of *P. acnes* (Figure S3) and USA300 (data not shown). It was reported that glycerol can be produced endogenously by the breakdown of triglycerides by sebaceous gland-associated lipase in human skin [38]. Bacteria also produce glycerol during fermentation of glucose to ethanol [39]. Aquaporin-3 functions as a glycerol transporter in mammalian skin [40]. It is known that glycerol helps maintain healthy skin integrity [19]. Aquaporin 3-deficient mice exhibit skin defects, including impairment of water holding capacity, barrier recovery, and wound healing [40]. Thus, glycerol naturally produced in skin maybe acts as a bi-functional molecule that serves as a humectant to promote a healthy skin barrier and a fermentation inducer to trigger the probiotic activity of skin commensals. Although propionic acid exerted antimicrobial activity against USA300 *in vitro* (Figure 3D) and *in vivo* (Figure 3F), we cannot exclude the possibility that a mixture of SCFAs and/or other secretory proteins [41] or metabolites in glycerol fermentation products of *P. acnes* also contributed to the anti-USA300 effects of fermented media (Figure 2B and Figure 3C). Propionic acid in lesser amounts may be produced by *P. acnes* in the TBS culture media in the absence of glycerol since TSB contains dextrose (2.5 g/l), a D-glucose monohydrate, as a carbon source for bacterial growth. The presence of propionic acid and/or other secretory proteins or metabolites may explain a slight suppression of UCA300 growth *in vitro* by undiluted and glycerol-free media of *P. acnes* (Figure 2C).

It has been reported that bacteria possess metabolic capabilities to respond to low external pH [42]. This adaptation permits the induction of genes involved in an acid-tolerance response and synthesis of a series of acid shock proteins that are protective for extreme acidic conditions [42]. Mutants defective in AtrB lack an acid tolerance response to propionic acid, indicating that AtrB protein is necessary for the full induction of acid resistance by exposure to propionic acid [43]. Inactivation of PhoP significantly increased bacterial resistance to propionic acid [43]. Recently, *S. aureus* gene profile expression was investigated in a condition of acidification [44]. Results revealed that the expression of several genes was affected by a variation in pH from 7.5 to 5.5. Among these genes, one can find genes involved in maintaining the intracellular pH, such as the urease operon (ure) and genes encoding intracellular chaperones (clpB). Thus, although our results demonstrate that fermented media of *P. acnes* decrease the intracellular pH of *S. aureus* (Figure 2C), the effects of long-term use of skin probiotics containing SCFAs as antimicrobials induces an acid tolerance/resistance response are unknown. Although SCFAs are normal human metabolites and theoretically less toxic, SCFAs at high doses may create an extremely acid solution, which causes skin irritation and/or corrosion [45].

Future work will include developing anti-*S. aureus* skin probiotics containing live *P. acnes*, glycerol and/or SCFAs. Even though skin immune cells may block the entrance of *P. acnes* into bloodstream, it is worth examining if live *P. acnes* in the anti-*S. aureus* skin probiotics can enter bloodstream when skin probiotics are applied onto the skin wounds for consecutive days. The application of bacterial-free fermented media or a fermentation inducer (e.g. glycerol) that did not affect the growth of *P. acnes* (Figure S3) may be a safer approach. A recent study using a multilocus sequence typing scheme (MLST) for *P. acnes* has suggested that pathogenic versus truly commensal lineages of *P. acnes* may exist [33], [46]. *P. acnes* type IA_1_ strains including ATCC6919 in this study were found predominantly both in healthy skin and acne vulgaris [33]. Several strains of *P. acnes* were only ever isolated from healthy skin, particularly CC72 (type II) and CC77 (type III) strains, although whether they truly represent commensal lineages still remains to be firmly established. Besides *P. acnes*, other *Propionibacterium* species including *P. granulosum* and *P. avidum* found in human skin have the capability of utilizing carbohydrates for anaerobic fermentation [47], [48]. Therefore, as far as safety is concerned, the use of skin commensal strains of *P. acnes* or other *Propionibacterium* species as anti-*S. aureus* skin probiotics will be included in future development.

In human healthy skin, an immune response is not generally mounted against commensal *P. acnes*. Mice can display a robust inflammatory response to *P. acnes* [49] since *P. acnes* bacteria do not reside naturally in the mouse skin. Although a mouse wound model recapitulated human *S. aureus* infection (Figure 5) and the effects of *P. acnes*-induced inflammatory responses on skin lesions were subtracted from controls, injection of *S. aureus* with glycerol into an *ex vivo* human skin explant [50] that harbors commensal *P. acnes* will be part of future studies. To support our hypothesis that deep-seated abscesses create an anaerobic microenvironment, thus facilitating P. acnes fermentation to interfere with S. aureus, future work will also include collecting various skin biopsies from patients with S. aureus infection. Impetigo, a superficial infection of the skin that bacteria have a chance to expose to oxygen, while cellulitis is a deep-seated infection that invaded bacteria live in an anaerobic microenvironment around the deeper layers (connective tissue) of skin [51]. The abundance of bacteria (*P. acnes* and *S. aureus*) and SCFAs including propionic acid in the skin infections of impetigo and cellulitis should be compared. The significance in this study includes 1) validating the effectiveness of *P. acnes* fermentation as anti-*S. aureus* skin probiotics; and 2) providing a novel approach to treat *S. aureus* infections, thereby benefiting the large community of patients with *S. aureus*/MRSA infections [11]. Microbial fermentation has been widely employed in the development of various products including yogurt, wine, and vinegar. The concept of fermentation of skin commensals against pathogens not only creates potentially a new skin industry but also generates a new area of investigation into the biological function of skin microbiome for promoting human health.

## Materials and Methods

### Ethics Statement

This study was carried out in strict accordance with the recommendations in the Guide for the Care and Use of Laboratory Animals of the National Institutes of Health (NIH). Experiments of using mice were performed at University of California, San Diego (UCSD). The UCSD ethics committee specifically approved this study under an approved Institutional Animal Care and Use Committee (IACUC) protocol (no. S10058).

### Culture of Microorganisms

USA300 or *M. luteus* (ATCC9341) was cultured on 3% tryptic soy broth (TSB) (Sigma, St. Louis, MO, USA) agar overnight at 37°C. *P. acnes* (ATCC6919) was cultured on Reinforced Clostridium Medium (RCM, Oxford, Hampshire, England) under anaerobic conditions using Gas-Pak (BD, Sparks, MD, USA) at 37°C. All microorganisms from a single colony were cultured in their media. Overnight cultures were diluted 1∶100 and cultured to an absorbance at 600 nm [optical density (OD)_600_] = 1.0. Microorganisms were harvested by centrifugation at 5,000 g for 10 min, washed with PBS, and suspended in PBS.

### Anti-USA300 Overlay Assay

The *P. acnes* (10^5^ CFU) or *M. luteus* was inoculated in two parallel 2-cm streaks on 1.5% agar (Oxoid. Ltd., London, UK) plates containing rich medium [10 g/l yeast extract (Biokar Diagnostics, Beauvais, France), 5 g/l TSB, 2.5 g/l K_2_HPO_4_ and 1.5 g/l KH_2_PO_4_] in the absence and presence of 20 g/l glycerol under anaerobic conditions using Gas-Pak (BD, Sparks, MD, USA) at 30°C for three days. Soft agar (1%) was cooled to 45°C before USA300 bacteria were added to obtain a concentration of (10^4^ CFU/ml). The soft agar (10 ml) was then poured onto the plates to overlay the rich medium agar. After incubation at 30°C for 40 h under anaerobic conditions, the plates were examined visually.

### Probiotic Effects of *P. acnes* Fermentation Against USA300

*P. acnes* (10^5^ CFU/ml) was incubated in rich medium (10 ml) in the absence and presence of 20 g/l glycerol under anaerobic conditions using Gas-Pak (BD, Sparks, MD, USA) at 30°C. Rich medium plus 20 g/l glycerol without *P. acnes* was included as a control. The 0.001% (w/v) phenol red (Sigma, St. Louis, MO, USA) in rich medium with 20 g/l glycerol served as an indicator, converting from red-orange to yellow when fermentation occurs. To assess the probiotic activity of fermentation products, *P. acnes* was incubated in phenol red-free rich medium with/without glycerol for seventeen days. After fermentation, *P. acnes* was discarded by centrifugation at 5,000 g for 30 min. Media were then passed through 0.2-*µ*m-pore-size filters. After filtration, the initial medium or its dilution (1/2 to 1/16) was added to USA300 (10^5^ CFU/ml in TSB) on a 96-well microplate overnight. The plates were mixed well and then OD_600_ was measured by a microplate reader to estimate bacterial growth.

### Measurement of Intracellular pH

Measurement of intracellular pH of USA300 using a cFSE florescence probe (Life Technologies, Grand Island, NY, USA) was previously described [52]. Briefly, USA300 bacteria were loaded with cFSE (5 µM) for 30 min at 37°C in 50 mM HEPES and 5 mM ethylenediaminetetraacetic acid (EDTA). To eliminate unbound probe, bacteria were incubated with glucose (10 mM) for an additional 30 min, washed twice in 50 mM PBS with 10 mM MgCl_2_, pH 7.0, and then re-suspended in 1 mM PBS. The cFSE-loaded bacteria (3×10^4^ CFU) were dispensed in on a 96-well microplate containing 100 µl/per well of rich medium plus 20 g/l glycerol, or culture supernatants of *P. acnes* in rich medium in the absence and presence of 20 g/l glycerol. Fluorescence intensities were measured immediately and every min for 5 min using an excitation wavelength of 490 nm and emission wavelength of 520 nm. A drop in relative fluorescence indicates the decrease in intracellular pH. Fluorescence of the bacteria-free filtrate (background fluorescence) was measured after the 5-min assay. In this case, treated suspensions were centrifuged at 5,000 g for 5 min. The fluorescence of the bacteria-free supernatant was measured and deducted from values for the treated suspensions. Calibration curves were obtained by incubation of un-treated, cFSE-loaded bacteria in buffers of various pHs. The buffer containing glycine (50 mM), citric acid (50 mM), Na_2_HPO_4_.2H_2_O (50 mM), and KCl (50 mM) was adjusted to various pH values ranging from 4 to 10. Equilibration of the intracellular and extracellular pH was conducted by addition of 1 µM valinomycin and nigericin (Sigma, St. Louis, MO, USA).

### *In vivo* Effects of Fermented Media and Propionic Acid on Skin Infection of USA300

ICR mice (2–3 month-old females; Harlan Labs, Placentia, CA, USA) were anesthetized by isoflurane. A 5 mm wound was made on the dorsal skin following shaving with electrical clippers. Following skin wounding, we applied 5 µl of media of *P. acnes* (10^5^ CFU/ml in 10 ml) incubated with/without glycerol or media plus glycerol (control) for 17 days to the wounded areas. To determine if propionic acid can prevent or mitigate the infection of CA-MRSA, 5 µl of 100 mM propionic acid was applied to the wounded areas. Application of PBS (pH 3.5, corresponding to pH for 100 mM propionic acid) or PBS (pH 7.1) to the wounded areas served as controls. The wounds were left uncovered throughout the experimental period. To simulate bacterial infection, USA300 (2×10^6^ CFU in 5 µl PBS) was applied to the wounds 10 min after application of media, propionic acid or PBS controls. To measure the extent of wound closure, a transparent parafilm was placed over the wounded skin and the area was marked by outlining the area of the wound. The lesion size (mm^2^) was measured daily for 8 days then calculated with ImageJ software (NIH, Bethesda, MD, USA). Five mice per group per experiments were used. We used a Student’s *t*-test to determine the significance of the differences between groups. Data represent the mean ± SD from three independent experiments. All experiments using mice were conducted in a biosafety level 2 (BSL-2) facility and in accordance with institutional guidelines for animal experiments. To determine the bacterial counts in infected skin, the infected skin was excised 72 h following bacterial application. The excised skin was weighted and homogenized in 200 µl of sterile PBS with a tissue grinder. Bacterial CFUs in the skin were enumerated by plating serial dilutions (1∶10^1^–1∶10^6^) of the homogenate on a TSB agar plate. The plate was incubated for 24 h at 37°C to count colonies. The bacterial numbers (CFUs) per gram of excised skin were calculated and presented as % of control.

### MBC Assays

To determine the MBC of propionic acid, bacteria (10^6^ CFU/ml) were incubated with propionic acid at various concentrations (5–100 mM in PBS) in media on a 96-well microplate (100 µl per well) overnight. The control received only PBS. After incubation, bacteria were diluted 1∶10–1∶10^6^ with PBS. MBC was finally examined at a 99.9% killing level and determined by spotting the dilution (5 µl) on an agar plate supplemented with media for the counting of CFUs.

### Validation of *P. acnes* Glycerol Fermentation *in vivo* by NMR Analysis

The ear of ICR mice was intradermally injected with ^13^C_3_-glycerol (0.2 mg) and P. acnes (ATCC6919; 10^7^ CFU in 10 µl PBS) for one, two or three days. The other ear of the same mouse received ^13^C_3_-glycerol (0.2 mg) and PBS (10 µl) as a control. Supernatants (500 µl) of ear homogenates were mixed with 10% D_2_O for NMR analysis. The 1-D NMR spectra were measured on a JEOL-ECS NMR spectrometer (JEOL USA, Inc., Peabody, MA, USA) operating at resonance frequency of 400 MHz with a repetition delay of 3 sec for ^13^C NMR. The 2-D ^1^H-^13^C heteronuclear single quantum correlation (HSQC) NMR spectra were acquired on a Bruker Avance 600 MHz NMR spectrometer (Bruker Daltonics Inc., Fremont, CA, USA) with a triple resonance inverse (TCI) cryo-probe and recorded as 2048×256 complex points with 32 scans and 1 sec repetition time. Newly appearing signals belong to the intermediates or final products resulting from ^13^C_3_-glycerol fermentation by P. acnes [53].

### Interference of *P. acnes* Fermentation with the Colonization of USA300 *in vivo*

A 5 mm wound made on the dorsal skin of ICR mice were described above. *P. acnes* (10^7^ CFU in 5 µl PBS), P. acnes and glycerol (0.2 mg), PBS (5 µl) alone or glycerol alone were applied onto the skin wounds for 3 days. USA300 (10^7^ CFU in 5 µl PBS) was administered onto the same wounds. The wounds were excised, weighted and homogenized 3 days after USA300 administration. Bacterial CFUs in the wounds were enumerated by plating serial dilutions (1∶10^1^–1∶10^6^) of the homogenate on a TSB agar plate. The plate was incubated for 24 h at 37°C to count colonies of USA300. After 24 h incubation, only USA300 colonies were formed. It normally takes more than 3 days for *P. acnes*, a relatively slow-growing bacterium, to form visible colonies on TSB agar plates.

### Statistical Analysis

To determine significances between groups, comparisons were made using the two-tailed *t*-test. For all statistical tests, the *P*-values of <0.05 (*), <0.01 (**), and <0.001 (***) were accepted for statistical significance.

## Supporting Information

Figure S1 **Validation of** ***P. acnes*** **glycerol fermentation via identification of SCFAs in the fermented media by NMR analysis.** Fermented media of *P. acnes* were centrifuged and passed through a 0.2 µm filter. Supernatants were then mixed with 10% D_2_O and analyzed by NMR spectrometers. Representative 1-D ^13^C- (A) and 1H- (B) NMR spectra (400 MHz JEOL JNM-ECS) that reveal the principal SCFAs in the fermented media seventeen days after addition of ^13^C_3_-glycerol. (C) A 2-D ^1^H-^13^C HSQC NMR spectrum (600 MHz) was displayed. In addition to glycerol (Gly), three SCFAs [acetic acid (Ac), lactic acid (L), and propionic acid (P)] were detected in the fermentation products of *P. acnes*.
(TIF)

Figure S2 **Suppression of USA300-infected lesions by propionic acid.** A 5-mm long excision wound will be created on the back of ICR mice. To assess if propionic acid alleviates the lesions caused by USA300 infection, USA300 bacteria (2×10^6^ CFU) were applied onto the wounded areas 10 min after application of propionic acid (5 µl; 100 mM) or PBS (5 µl). (A) Skin lesions were pictured on day 1 after bacterial application. (B) Inflammation (arrows) surrounding the skin lesions (▾) was observed in the H&E-stained frozen sections [low (upper panels) and high (lower panels) powers] of skins applied with USA300 and controls. The scale bars of low power and high power were 40 µm, respectively.
(TIF)

Figure S3 **Comparison of growth curves of** ***P. acnes*** **in the absence of presence of glycerol.** *P. acnes* was incubated in rich medium in the absence (□) and presence (▪) of glycerol on a 96-well microplate under anaerobic conditions at 30°C. The OD_600_ was read at the indicated time points. Data are the mean ± SD of three separate experiments. Glycerol did not change significantly in the growth of *P. acnes*.
(TIF)

Methods and Materials S1 **Confirmation of** ***P. acnes*** **fermentation by NMR analysis.**
(DOCX)
