# Supplementary figures and images for: Fermentation of Propionibacterium acnes, a Commensal Bacterium in the Human Skin Microbiome, as Skin Probiotics against Methicillin-Resistant Staphylococcus aureus
Source: PLoS One. 2013 Feb 6;8(2):e55380. doi: 10.1371/journal.pone.0055380 (PMC3566139; doi:10.1371/journal.pone.0055380)

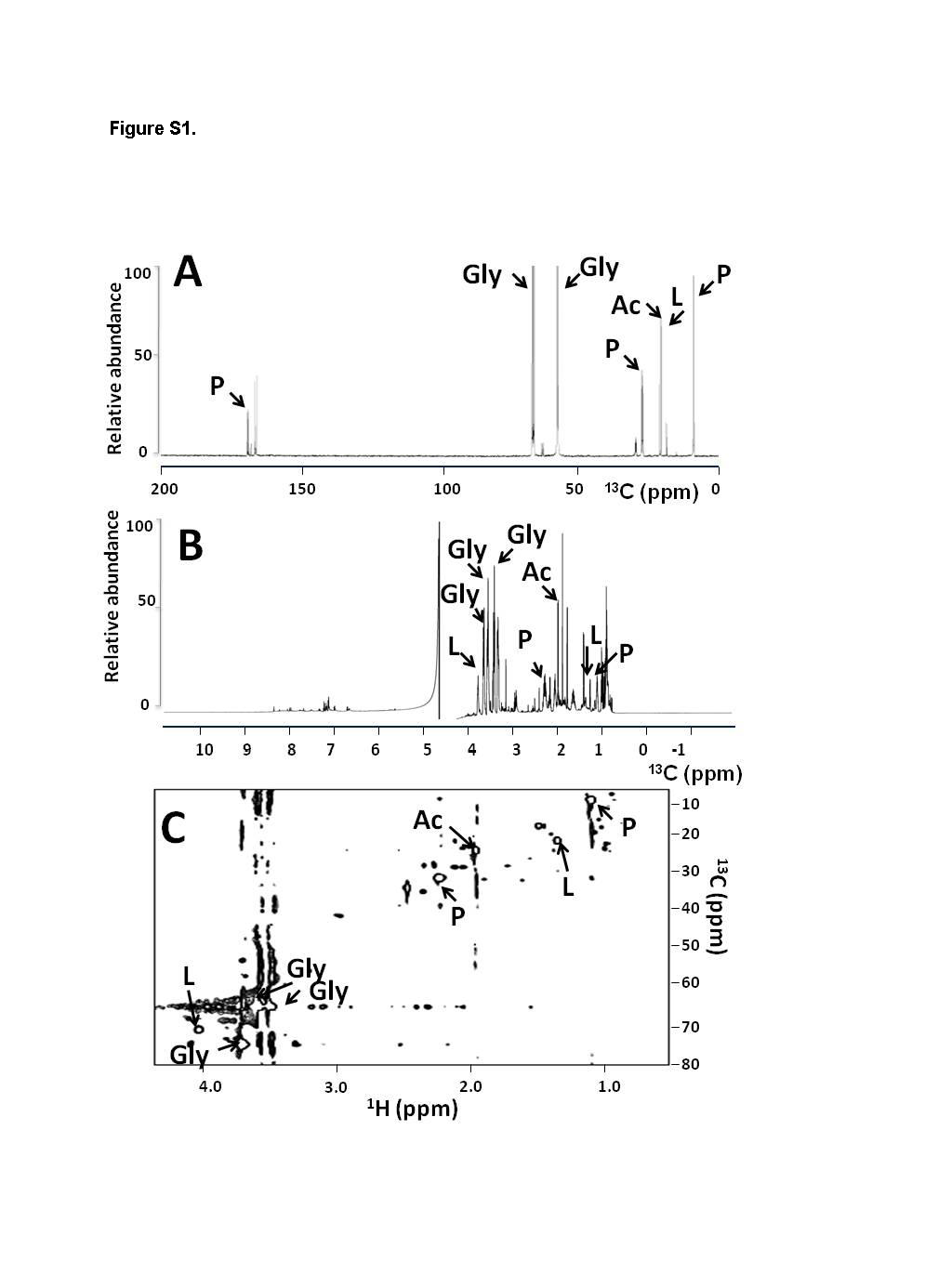

Supplement: Figure S1 — Validation of P. acnes glycerol fermentation via identification of SCFAs in the fermented media by NMR analysis. Fermented media of P. acnes were centrifuged and passed through a 0.2 µm filter. Supernatants were then mixed with 10% D2O and analyzed by NMR spectrometers. Representative 1-D 13C- (A) and 1H- (B) NMR spectra (400 MHz JEOL JNM-ECS) that reveal the principal SCFAs in the fermented media seventeen days after addition of 13C3-glycerol. (C) A 2-D 1H-13C HSQC NMR spectrum (600 MHz) was displayed. In addition to glycerol (Gly), three SCFAs [acetic acid (Ac), lactic acid (L), and propionic acid (P)] were detected in the fermentation products of P. acnes. (TIF) [file pone.0055380.s001.tif]

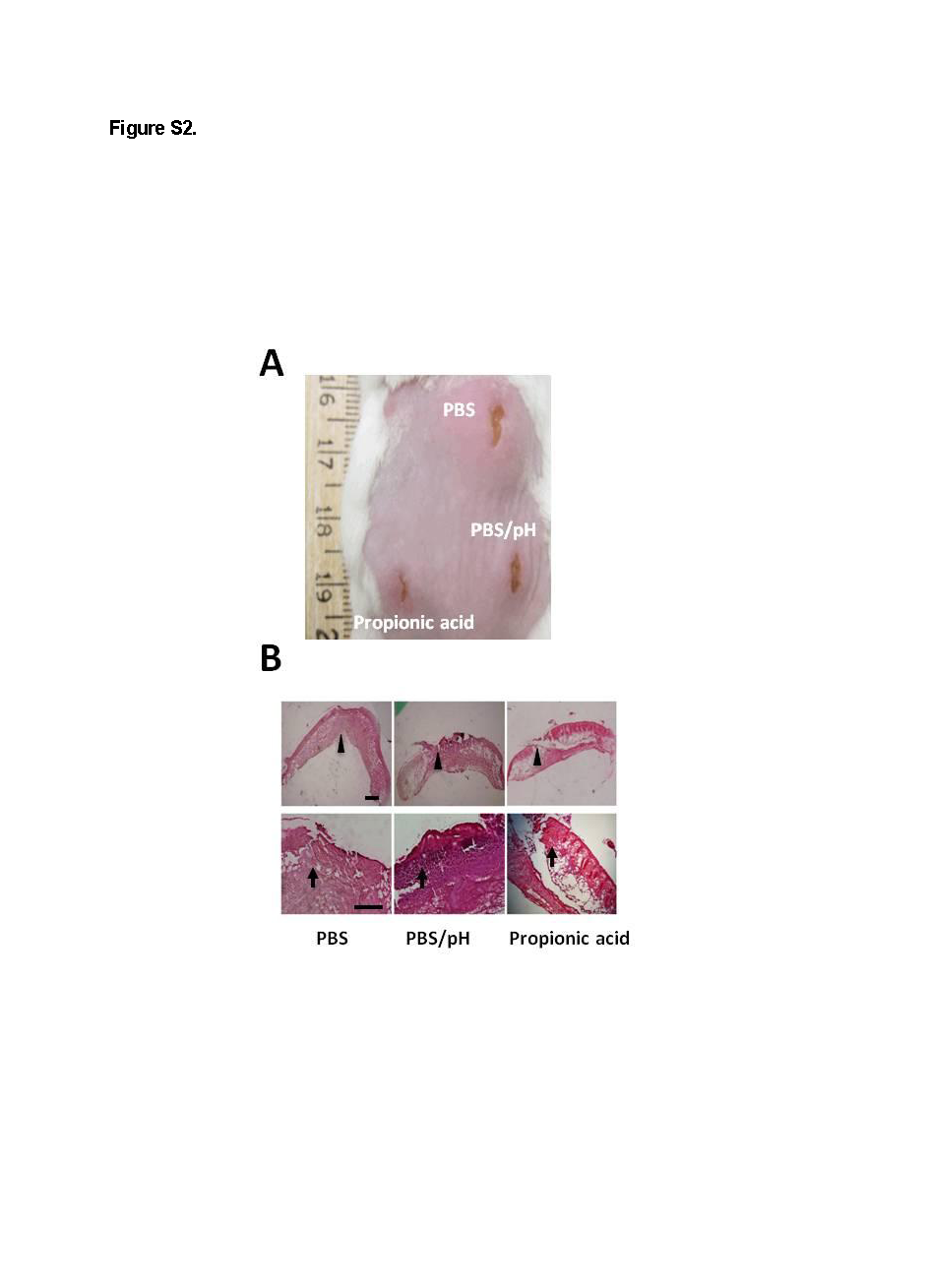

Supplement: Figure S2 — Suppression of USA300-infected lesions by propionic acid. A 5-mm long excision wound will be created on the back of ICR mice. To assess if propionic acid alleviates the lesions caused by USA300 infection, USA300 bacteria (2×10^6^ CFU) were applied onto the wounded areas 10 min after application of propionic acid (5 µl; 100 mM) or PBS (5 µl). (A) Skin lesions were pictured on day 1 after bacterial application. (B) Inflammation (arrows) surrounding the skin lesions (▾) was observed in the H&E-stained frozen sections [low (upper panels) and high (lower panels) powers] of skins applied with USA300 and controls. The scale bars of low power and high power were 40 µm, respectively. (TIF) [file pone.0055380.s002.tif]

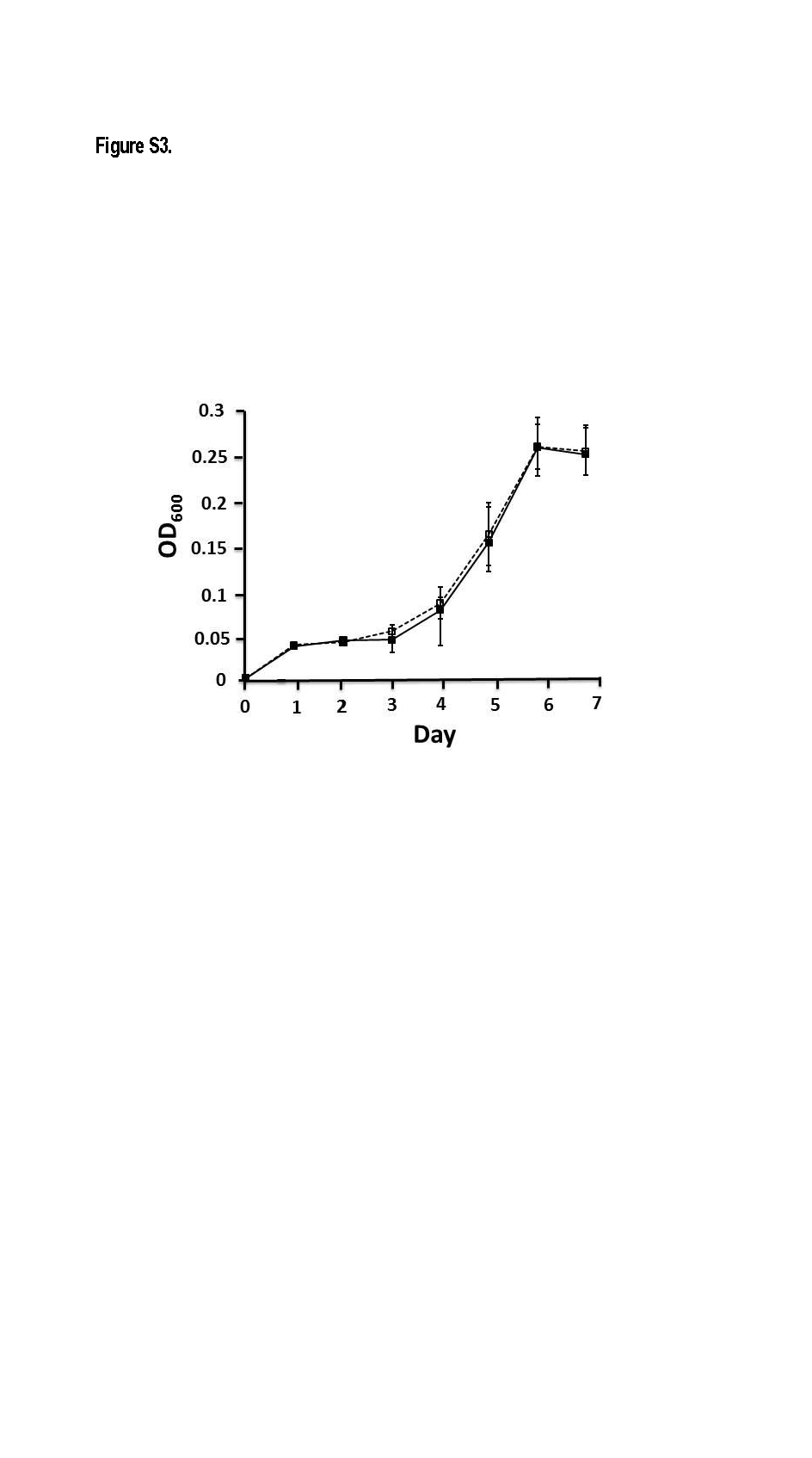

Supplement: Figure S3 — Comparison of growth curves of P. acnes in the absence of presence of glycerol. P. acnes was incubated in rich medium in the absence (□) and presence (▪) of glycerol on a 96-well microplate under anaerobic conditions at 30°C. The OD600 was read at the indicated time points. Data are the mean ± SD of three separate experiments. Glycerol did not change significantly in the growth of P. acnes. (TIF) [file pone.0055380.s003.tif]
