# Supplementary material for: Fermentation of Propionibacterium acnes, a Commensal Bacterium in the Human Skin Microbiome, as Skin Probiotics against Methicillin-Resistant Staphylococcus aureus
Source: PLoS One. 2013 Feb 6;8(2):e55380. doi: 10.1371/journal.pone.0055380 (PMC3566139; doi:10.1371/journal.pone.0055380)
Supplement: Methods and Materials S1 — Confirmation of P. acnes fermentation by NMR analysis. (DOCX) [file pone.0055380.s004.docx]

**Methods and Materials S1. Confirmation of *P. acnes* fermentation by NMR analysis.**

To validate the fermentation of *P. acnes*, bacteria (10^5^ CFU/ml) were incubated in (10 ml) rich media with (20 g/l) ^13^C_3_-glycerol (Cambridge Isotope Laboratories, Andover, MA, USA) under anaerobic conditions using Gas-Pak (BD, Sparks, MD, USA) at 30°C. After seventeen-incubation, *P. acnes* was discarded by centrifugation at 5,000 g for 30 min. Supernatants were then passed through 0.2-μm-pore-size filters and mixed with 10% D_2_O for NMR analysis. The 1-D NMR and 2-D ^1^H-^13^C HSQC NMR spectra were obtained according to methods as described in Materials and Methods. The previously published NMR spectra of ^13^C-glycerol fermentation by *Propionibacterium* species were used to assist in identifying the intermediates or final products resulting from ^13^C_3_-glycerol fermentation by *P. acnes* [53].

**Histological analysis of USA300-infected skin**

The USA300-infected skins pre-treated with propionic acid and its controls were cross-sectioned, stained with H&E (Sigma, St. Louis, MO, USA), and viewed on a Bx51 research microscope (Olympus, Melville, NY, USA).

**The effect of glycerol on the growth of *P. acnes***

*P. acnes* (10^5^ CFU/ml) was incubated in rich medium (100 µl/well) in the absence and presence of 20 g/l glycerol on a 96-well microplate under anaerobic conditions using Gas-Pak (BD, Sparks, MD, USA) at 30°C. Reading 96-well microplates at OD_600_ was used to determine growth of *P. acnes*.
